# Subdural Hæmorrhage with Convulsions

**Published:** 1901-06-08

**Authors:** 


### Hospital Clinics and Medical Progress.

### SUBDURAL HÆMORRHAGE WITH CONVULSIONS.

At the last meeting of the Clinical Society a case was related by Dr. Risien Russell and Mr. Raymond Johnson in which convulsions, occurring on the sixth day after an injury to the head, were successfully treated by operation. A man, thirty-three years of age, fell whilst intoxicated down a flight of stone steps. The immediate symptoms were those of concussion, and there was a superficial scalp wound in the left parietal region. During the next five days there were no noticeable symptoms, but on the sixth day convulsions supervened, and these were frequently repeated. They involved the left side of the body, spreading across to the right side of the face and the right leg, and in some of the most severe fits to the right arm also. There was a strong family history of epilepsy, and the urine contained albumin. In consequence, however, of the distribution of the convulsions, presence of the extensor response, and of marked left sided paresis between the fits, the existence of an irritative lesion of the right motor area of the cortex was diagnosed. The inhalation of chloroform and the administration of chloral and bromide of potassium having failed, operation was decided upon. The trephine was applied over the right Rolandic area, and several drachms of dark blood were evacuated on incising the dura mater. The surface of the brain presented no unusual appearances beyond a very slight bulging. No convulsions occurred after the operation; the paresis of the left side became gradually less; and the muscular power became perfect at the end of a month. No satisfactory explanation of the long delay in the onset of the symptoms could be given, it being considered that, in the absence of all evidence of laceration of the brain substance, they could hardly be regarded as the result of spreading cerebral œdema.

